# Theoretical attributable risk analysis and Disability Adjusted Life Years (DALYs) based on increased dairy consumption

**DOI:** 10.1186/s12889-022-14042-7

**Published:** 2022-08-27

**Authors:** Sarah S. Cohen, Lauren C. Bylsma, Naimisha Movva, Dominik D. Alexander

**Affiliations:** 1 EpidStrategies, a division of ToxStrategies, Inc., 1249 Kildaire Farm Road #134, Cary, NC 27511 USA; 2 MetaMethod, 13570 Summit Circle, Poway, CA 92064 USA

**Keywords:** Dairy, Preventive Fraction, Disability Adjusted Life Years, DALYs, Attributable Fraction, Chronic Disease

## Abstract

**Background:**

Identification of modifiable risk factors that may impact chronic disease risk is critical to public health. Our study objective was to conduct a theoretical population attributable risk analysis to estimate the burden of disease from low dairy intake and to estimate the impact of increased dairy intake on United States (US)-based disability adjusted life years (DALYs).

**Methods:**

We conducted a comprehensive literature review to identify statistically significant summary relative risk estimates (SRREs) from recent meta-analyses of dairy consumption and key chronic disease outcomes. The SRREs were applied to preventive fractions using a range of categories (low to high) for population consumption of dairy products. The preventive fraction estimates were then applied to the number of DALYs for each health outcome in the US based on 2019 WHO estimates. The population attributable risk proportion estimates were calculated using the inverse of the SRRE from each meta-analysis using the same range of categories of consumption. These values were subsequently applied to the DALYs estimates to estimate the theoretical burden of disease attributable to low dairy intake.

**Results:**

Statistically significant SRREs were identified in recent meta-analyses of total dairy consumption in relation to breast cancer, colorectal cancer, cardiovascular disease (CVD), type 2 diabetes (T2D), stroke, and hypertension. In this theoretical analysis, nearly 850,000 DALYs (or 5.0% of estimated years of healthy life lost) due to CVD and 200,000 DALYs (4.5%) due to T2D may be prevented by increased dairy consumption. Approximately 100,000 DALYs due to breast cancer (7.5%) and approximately 120,000 DALYs (8.5%) due to colorectal cancer may be prevented by high dairy intake. The numbers of DALYs for stroke and hypertension that may be prevented by increased dairy consumption were approximately 210,000 (6.0%) and 74,000 (5.5%), respectively.

**Conclusions:**

Consumption of dairy products has been associated with decreased risk of multiple chronic diseases of significant public health importance. The burden of disease that may potentially be prevented by increasing dairy consumption is substantial, and population-wide improvement in meeting recommended daily dairy intake goals could have a notable public health impact. However, this analysis is theoretical, and thus additional studies providing empirical evidence are needed to further clarify potential relationships between dairy intake and various health outcomes.

**Supplementary Information:**

The online version contains supplementary material available at 10.1186/s12889-022-14042-7.

## Background

With the rising burden of chronic disease on a global scale, the identification of modifiable factors that may impact disease risk is paramount. Several behaviors, such as cigarette smoking, low physical activity, and excess body weight, have been identified as important risk factors for multiple chronic diseases [1–4]. Although diet and nutrition have been studied extensively with respect to chronic disease, including cardiovascular disease (CVD), type 2 diabetes (T2D), and cancer, relatively few dietary factors have been established as contributing to risk of these outcomes. However, higher intake of dairy products has been associated with decreased risks of several chronic diseases in multiple prospective cohort studies and meta-analyses [5–9] and was summarized in a recent umbrella review [10].

Dairy products provide an important source of key nutrients, including whey proteins, casein proteins, calcium, and essential vitamins and minerals. Calcium and magnesium found in dairy products are crucial factors in insulin response and glucose tolerance, and whey proteins can affect glycemic control. Milk proteins have been shown to decrease ghrelin levels and improve satiety and appetite control and prevent weight gain [11]. The vitamins, minerals, and proteins present in dairy products may provide beneficial effects on inflammatory biomarkers, oxidative stress, blood pressure, and CVD [5, 12, 13]. The active metabolite of vitamin D, 1,25(OH)_2_D_3_, has been shown to target cellular processes such as apoptosis, cellular adhesion, and cell cycle progression in several lines of cancer cells [14].

Despite these findings, some controversy remains regarding the association between dairy products and chronic disease, mainly due to the presence of saturated fatty acids in milk fat. Evidence from experimental studies indicates that consumption of saturated fats increases the amount of plasma total cholesterol, LDL cholesterol, and apolipoprotein B levels, which have been associated with increased risk of heart disease [15, 16], but not all studies support an association between saturated fat intake and CVD [17, 18]. Some epidemiological studies have observed an increased risk of breast cancer with high levels of saturated fatty acid intake [19, 20]. However, large comprehensive meta-analyses have indicated that dairy products reduce the risk of several adverse health outcomes [5–9].

The United States Department of Agriculture (USDA) Dietary Guidelines for Americans 2020 recommends an equivalency of 3 cups of dairy per day for adults [21]. However, the average American consumes approximately 1.53 servings of dairy per day, with men and women over the age of 20 consuming 1.64 and 1.27 servings, respectively, according to the National Health and Nutrition Examination Survey 2017–2018 data [22]. The groups with the lowest average daily intake are men aged 60–69 and women aged 70 and above, with an average intake of 1.35 servings per day for men and 1.21 for women [22]. The per capita annual average intake of all dairy products decreased from 703 pounds in the 1950s to 593 pounds in 2000. During that time, the per capita annual average consumption of cheese more than quadrupled, while the consumption of milk decreased by 38% [23]. The Scientific Report of the 2020 Dietary Guidelines Advisory Committee reported that only 11.65% of Americans aged ≥ 1 year met or exceeded the recommendation for dairy consumption [24].

Given the beneficial role of dairy intake on chronic disease risk and the fact that most Americans are not meeting recommended intake levels, our objective was to construct theoretical attributable risk models to estimate the potential impact that dairy consumption modification could have on chronic disease in the general US population, using burden of disease data from the World Health Organization (WHO).

Under the health statistics and information systems program of the WHO [25], the burden of disease from mortality and morbidity are quantified for health decision making and planning. For example, in 2009 the WHO published a report entitled, *Global Health Risks: Mortality and Burden of Disease Attributable to Selected Major Risks* [2] describing diseases and injuries and the risk factors that cause them. Subsequent to the WHO report, articles were published in Lancet [26] and the Journal of the American Medical Association [27] that reported on the risk assessment of the burden of disease, injuries, and risk factors. These two articles utilized attributable risk methodology in the context of disability adjusted life years (DALYs). DALYs are a metric for burden of disease that was conceived and developed by the WHO [28]. One DALY can be thought of as one lost year of “healthy” life, and these values can be summed at the population level to produce an estimate of the global burden of disease attributable to specific factors. In the US specifically, dietary risk factors was the third leading cause of DALYs after tobacco use and high body mass index in 2016 among the seventeen risk factors identified in the study [27], indicating that a prominent modifiable risk factor such as diet can significantly impact the population-level burden of disease.

Therefore, using the concept of attributable risk and burden of disease on a public health scale, we conducted theoretical preventive fraction and population attributable risk analyses of total dairy intake and selected health and disease outcomes. Data from these analyses were applied to US WHO DALYs figures to estimate the impact on burden of disease.

## Materials and methods

### General overview

**Fig. 1:**
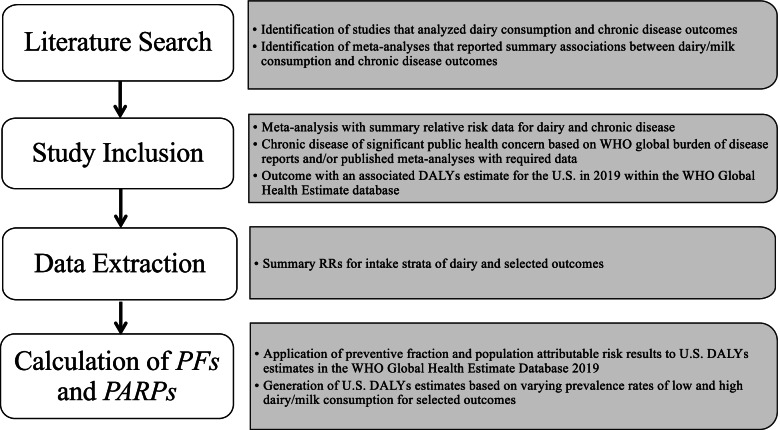
Overview of methodological and analytical protocol

In 2012, Doidge et al. [29] published an attributable risk analysis pertaining to health care savings from increased consumption of dairy products. The results from their attributable risk analysis were applied to economic and disease burden information for Australia. Specifically, the authors identified risk estimates for the association between dairy consumption and certain health outcomes such as stroke and hypertension and estimated the DALYs and economic savings of increased dairy intake. Using similar methodology, we conducted a literature search to identify all possible outcomes significantly associated with dairy intake, extracted the most relevant summary relative risk estimates from comprehensive meta-analyses, conducted preventive fraction and population attributable risk analyses, and applied results to US-based DALYs data. Our methodological and analytical approach is illustrated in Fig. 1.

### Literature search and study inclusion

We implemented a two-phase literature search and study inclusion protocol. In the first phase, we conducted a series of comprehensive literature searches in May 2021 to identify studies that reported risk estimates (regardless of direction of effect) for dairy intake and chronic disease outcomes (e.g., heart disease, T2D, cancer) (Supplemental Table 1). For this phase, our focus was on published meta-analyses as the objective was to include the most comprehensive estimate of the association between dairy intake and chronic disease outcomes. Meta-analyses were included under the following criteria: i) the study analyzed prospective studies; ii) the study reported a summary relative risk estimate for high versus low total dairy consumption associated with the health or disease outcome; iii) the outcome was of considerable public health significance (based on review of the WHO global burden of disease reports) and iv) the adverse health outcome had a DALYs estimate for the United States in 2019 within the WHO Global Health Estimates database. If multiple meta-analysis publications were identified for the same topic area, the most recently published or most analytically comprehensive meta-analysis was selected for the analysis.

In the second phase, we identified risk estimates for total dairy (a composite variable for all dairy intake), total milk, and low-fat or high-fat dairy products from the articles identified in the first phase. Analyses of other specific dairy products (e.g., yogurt, cheese) were not included as the data were more limited and variable across studies. Our objective was to calculate preventive fraction and population attributable risk percentages; the informativeness of such estimates is based upon established or causal relationships. We did not conduct systematic causation analyses nor are we specifically implying that causal relationships exist. Rather, the purpose of this assessment is to provide a theoretical framework for population-based attributable risk analyses for a dietary intake variable (i.e., dairy) that has been consistently associated with decreased chronic disease risk across numerous analytical epidemiologic investigations. Thus, as a conservative approach, we selected only topic areas for which statistically significant meta-analysis findings between dairy intake and chronic disease were reported. In addition, studies with reported statistically significant summary associations for total milk intake were included in our assessment. No meta-analyses were identified that observed consistently statistically significant increased risks of disease based on dairy consumption.

### Analytical methodology

From each meta-analysis, we extracted summary relative risks for the highest vs. lowest intake strata of total dairy or total milk consumption and the outcomes of interest. These risk estimates were then used to calculate preventive fractions and population-attributable risk proportions (PARPs).

For exposures that decrease disease risk, a preventive fraction (or proportion) can be calculated to estimate the proportion of disease in the population that may be prevented if the population was exposed or achieved a certain level of exposure to a factor [30]. The formula for calculating the preventive fraction is *Pr (1 – RR)*, where *Pr* = prevalence of exposure and *RR* = relative risk for disease associated with exposure. In nutritional epidemiology studies, intake categories often vary considerably between studies, and distinctions made between intake strata may be arbitrary and may not represent thresholds in either the distribution of consumption or the relationship to disease risk [29]. Therefore, we used a conservative range of prevalence exposures (*Pr* of 15%, 25%, 35%, and 50%) to represent the proportion of the general population who consume high levels of dairy. Importantly, the designation of ‘high’ dairy is based on the highest intake strata across the studies and does not differ appreciably from the USDA recommended daily intake levels for dairy. The highest intake categories across the nutritional epidemiology studies typically range between three and four servings per day. Therefore, our estimates are based on “high” categories of dairy intake in the epidemiology studies which are also in line with USDA recommendations. As the current proportion of Americans that meet the USDA recommendations for dairy intake is approximately 11.65% [24], the prevalence exposures used in the analyses represent a range of increased population-level dairy consumption levels in the US compared with the current prevalence.

For exposures that increase disease risk, a PARP can be calculated to estimate the proportion of disease that may be attributed to the exposure. Since all summary estimates for dairy were in the inverse direction (i.e., decreased risk of disease in relation to high dairy intake), we used the inverse of the summary RRs from the selected meta-analyses to estimate disease risk based on low dairy consumption (vs. the high intake strata) and the proportion of disease therefore attributable to low dairy intake. The same four prevalence proportions were used to represent dairy intake levels in the general population. The following formula was used to calculate the PARP estimates:

$$\text{Population}\ \text{attributable}\ \text{risk}\ \text{proportion}= \frac{Pr \left({RR}_{I}-1\right)}{\left[1+Pr\left({RR}_{I}-1\right)\right]}$$

where *Pr* = prevalence of exposure and

*RR*_*1*_ = the inverse of the meta-analysis summary risk estimates.

The preventive fraction and PARP estimates for each dairy-outcome were applied to WHO global burden of disease data. Specifically, the disability-adjusted life year (DALY) estimates for the United States by cause were selected from the WHO Global Health Estimate Database for 2019 [31]. In its technical report, the WHO describes the DALY as “a summary measure which combines time lost through premature death and time lived in states of less than optimal health, loosely referred to as ‘disability’” [28]. The DALY is age-adjusted, does not discount for time, and is calculated using the following formula:

$DALY\left(c,s,a,t\right) = YLL\left(c,s,a,t\right) +YLD\left(c,s,a,t\right)$ for given cause *c*, age *a*, sex and year *t.*

The formula for years of life lost (YLL) for a given cause *c*, age *a*, sex *s* and year *t* is the following:

$$YLL\left(c,s,a,t\right)=N\left(c,s,a,t\right) x L\left(s,a\right)$$

where *N(c,s,a,t*) is the number of deaths due to the cause *c* for the given age *a* and sex *s* in year *t,*

*L(s,a)* is a standard loss function specifying years of life lost for a death at age *a* for sex *s* using highest projected life expectancies for 2050.

The formula for years lived with disability (YLD) is adjusted for comorbidity and calculated using the following formula:

$$YLD\left(c,s,a,t\right)=I\left(c,s,a,t\right) x DW\left(c,s,a\right) x L \left(c,s,a,t\right)$$

where *I(c,s,a,t)* is the number of incident cases for cause *c*, age *a* and sex *s,*

*DW(c,s,a)* is the disability weight for cause *c*, age *a* and sex *s* on a scale from 0 (perfect health) to 1 (dead) and,

*L(c,s,a,t)* is the average duration of the case until remission or death.

To estimate the number of DALYs in the US theoretically prevented by high consumption of total dairy or milk, we multiplied the preventive fractions for each outcome and prevalence exposure by the US 2019 DALY estimate for that specific factor provided by the WHO. To estimate the number of DALYs in the US attributable to low consumption of total dairy or milk, we multiplied the PARP estimates for each outcome and prevalence exposure by the US 2019 DALY estimate for that specific factor.

## Results

Our review of the literature yielded six chronic diseases with at least one published meta-analysis demonstrating a significant association with total dairy consumption: breast cancer [32], colorectal cancer [33], CVD [34], stroke [34], hypertension [35], and T2D [36]. The meta-analyses we considered for the six outcomes are provided in supplemental Table 2. Although we also identified several meta-analyses with statistically significant SRREs for other health outcomes, including osteoporosis and obesity, the WHO Global Burden of Disease report did not provide DALYs estimates for these outcomes and thus they were not included in our analysis. The overall summary relative risk estimates for high vs. low total dairy intake and the source of data including funding source are shown in Table 1. Each of these SRREs was applied to the preventive fraction and PARP calculations.

**Table 1:** Effect estimates of selected meta-analyses of total dairy consumption, milk consumption, low-fat dairy consumption, high-fat dairy consumption, and disease outcomes

Source	Disease Outcome	Studies Included in Analysis (n)	Comparison	RR	95% CI	*P*-value for Heterogeneity	I^2^ value	Funding Source
**Total Dairy Consumption**								
Dong (2011) [32]	Breast Cancer	10	High vs. Low	0.85	0.76–0.95	0.012	54.5%	National Natural Science Foundation of China
Schwingshackl (2018) [33]	Colorectal Cancer	18	High vs. Low	0.83	0.76–0.89	< 0.00	61%	None
Gholami (2017) [34]	Cardiovascular Disease	10	High vs. Low	0.90	0.81–0.99	0.009	55.8%	None
Schwingshackl (2017) [36]	Type 2 Diabetes	21	High vs. Low	0.91	0.85–0.97	< 0.0001	63%	NHS BRC grant (Interventional Public Health)
Gholami (2017) [34]	Stroke	16	High vs. Low	0.88	0.82–0.95	0.000	63.1%	None
Schwingshackl (2017) [35]	Hypertension	9	High vs. Low	0.89	0.86–0.93	0.65	0%	None
**Milk Consumption**								
Jin (2020) [37]	Colorectal Cancer	20	High vs Low	0.81	0.76–0.86	0.138	23.6%	National Research Foundation of Korea
Tian (2017) [38]	Type 2 Diabetes	7	High vs. Low	0.87	0.78–0.96	0.01	52.2%	National Natural Science Foundation of China; Wu Lian De Grant of Harbin Medical University
**Low-Fat Dairy Consumption**								
Dong (2011) [32]	Breast Cancer	4	High vs. Low	0.84	0.73–0.96	0.07	53.7%	National Natural Science Foundation of China
Aune (2013) [39]	Type 2 Diabetes	9	High vs. Low	0.83	0.76–0.90	0.67	0.0%	Liaison Committee between the Central Norway Regional Health Authority and the Norwegian University of Science and Technology
Gholami (2017) [34]	Stroke	9	High vs. Low	0.94	0.90–0.98	0.61	0.0%	None
Ralston (2012) [40]	Hypertension	4	High vs. Low	0.84	0.74–0.95	NR	38%	National Health and Medical Research Council, Australia
**High-Fat Dairy Consumption**								
Barrubes (2019) [41]	Colorectal Cancer	2	High vs. Low	0.68	0.53–0.87	0.06	71%	Interprofessional Dairy Organization (NLAC), Spain
Alexander (2016) [42]	Stroke	3	High vs. Low	0.91	0.84–0.99	0.882	0.0%	Dairy Research Institute

The results of the analyses of preventive fraction estimates and population attributable risk proportion estimates for the range of total dairy prevalence exposures are presented in Table 2. The preventive fraction estimates for the highest prevalence of population total dairy consumption ranged from as low as 4.5% of DALYs due to T2D and as high as 8.5% of DALYs due to colorectal cancer. For the lowest prevalence of population total dairy consumption, the preventive fraction estimates ranged from 1.35 to 2.55%. The PARP estimates demonstrated that between 1.5% (*Pr 15%,* T2D) and 8.5% (*Pr 50%,* colorectal cancer) of DALYs due to these chronic diseases may be attributable to low population total dairy consumption.

**Table 2:** Preventive fraction and population attributable risk proportion (PARP) estimates for total dairy, milk, low-fat dairy, and high-fat dairy consumption

Disease	Preventive Fraction				PARP			
	***Pr 15%***	***Pr 25%***	***Pr 35%***	***Pr 50%***	***Pr 15%***	***Pr 25%***	***Pr 35%***	***Pr 50%***
**Dairy Consumption**								
Breast Cancer	2.25%	3.75%	5.25%	7.50%	2.58%	4.23%	5.82%	8.11%
Colorectal Cancer	2.55%	4.25%	5.95%	8.50%	2.98%	4.87%	6.69%	9.29%
Cardiovascular Disease	1.50%	2.50%	3.50%	5.00%	1.64%	2.70%	3.74%	5.26%
Type 2 Diabetes	1.35%	2.25%	3.15%	4.50%	1.46%	2.41%	3.35%	4.71%
Stroke	1.80%	3.00%	4.20%	6.00%	2.00%	3.30%	4.56%	6.38%
Hypertension	1.65%	2.75%	3.85%	5.50%	1.82%	3.00%	4.15%	5.82%
**Milk Consumption**								
Colorectal Cancer	2.85%	4.75%	6.65%	9.50%	3.40%	5.54%	7.59%	10.50%
Type 2 Diabetes	1.95%	3.25%	4.55%	6.50%	2.19%	3.60%	4.97%	6.95%
**Low-Fat Dairy Consumption**								
Breast Cancer	2.40%	4.00%	4.80%	8.00%	2.78%	4.55%	6.25%	8.70%
Type 2 Diabetes	2.55%	4.25%	5.95%	8.50%	2.98%	4.87%	6.69%	9.29%
Stroke	0.90%	1.50%	2.10%	3.00%	0.95%	1.57%	2.19%	3.09%
Hypertension	2.40%	4.00%	5.60%	8.00%	2.78%	4.55%	6.25%	8.70%
**High-Fat Dairy Consumption**								
Colorectal Cancer	4.80%	8.00%	11.20%	16.00%	6.59%	10.53%	14.14%	19.05%
Stroke	1.35%	2.25%	3.15%	4.50%	1.46%	2.41%	3.35%	4.71%

*Pr* Prevalence exposure. Pr values of 15%, 25%, 35%, and 50% represent the proportion of the general population who consumes high levels of dairy

**Fig. 2:**
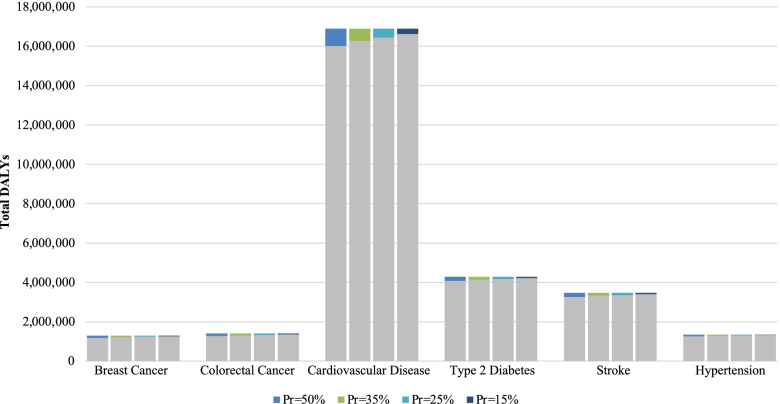
DALYs Attributable due to Low US Population-Level Dairy Consumption at Varying Exposure Prevalences. Gray bars represent the total number of US DALYs for each outcome of interest. Colored bar portions indicate the DALYs that may be attributable to lower population dairy consumption

**Fig. 3:**
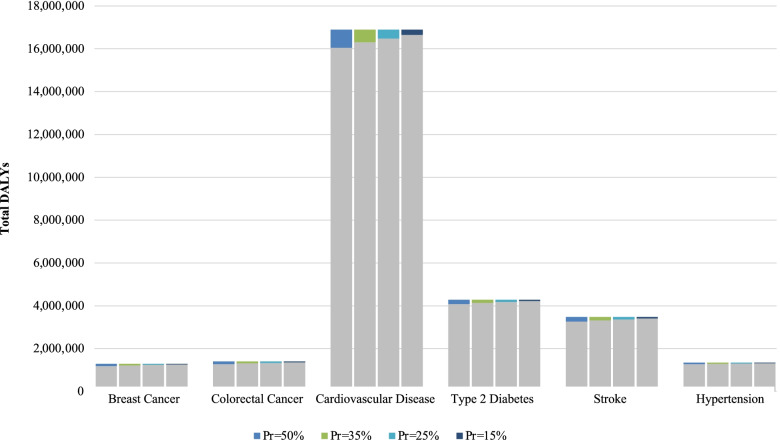
Preventable DALYs associated with High US Population-Level Dairy Consumption at Varying Exposure Prevalences. Gray bars represent the total number of US DALYs for each outcome of interest. Colored bar portions indicate the DALYs that may be preventable with higher population dairy consumption

The numbers of DALYs that could potentially be prevented by higher population consumption of total dairy and attributable to low population consumption of total dairy are presented in Table 3. The most significant impact of total dairy on DALYs was observed for CVD, mainly due to the high number of estimated DALYs for CVD in the US (about seventeen million). At a prevalence exposure of 50% for CVD, nearly 900,000 DALYs may be attributable to a low population total dairy intake (Fig. 2) and about 850,000 DALYs may be prevented by a higher population total dairy intake (Fig. 3).

**Table 3:** Preventable and attributable DALYs estimates for disease outcome and total dairy and milk consumption

Disease	US DALYs estimates 2019	DALYs Preventable with High Dairy Intake				DALYs Attributable to Low Dairy Intake			
		***Pr 15%***	***Pr 25%***	***Pr 35%***	***Pr 50%***	***Pr 15%***	***Pr 25%***	***Pr 35%***	***Pr 50%***
**Dairy Consumption**									
Breast Cancer	1,292,000	29,070.0	48,450.0	67,830.0	96,900.0	33,318.1	54,591.5	75,157.9	104,756.8
Colorectal Cancer	1,397,600	35,638.8	59,398.0	83,157.2	118,796.0	41,658.4	68,077.9	93,487.6	129,831.7
Cardiovascular Disease	16,900,400	253,506.0	422,510.0	591,514.0	845,020.0	277,055.7	456,767.6	632,635.3	889,494.7
Type 2 Diabetes	4,275,100	57,713.9	96,189.8	134,665.7	192,379.5	62,494.7	103,152.5	143,033.1	201,444.5
Stroke	3,473,500	62,523.0	104,205.0	145,887.0	208,410.0	69,624.7	114,511.0	158,228.9	221,712.8
Hypertension	1,342,400	22,149.6	36,916.0	51,682.4	73,832.0	24,434.2	40,235.4	55,662.3	78,129.1
**Milk Consumption**									
Colorectal Cancer	1,397,600	39,831.6	66,386.0	92,940.4	132,772.0	47,503.4	77,418.1	106,035.8	146,709.4
Type 2 Diabetes	4,275,100	83,364.5	138,940.8	194,517.1	277,881.5	93,720.6	153,951.0	212,470.8	297,199.5
**Low-Fat Dairy Consumption**									
Breast Cancer	1,292,000	31,008.0	51,680.0	62,016.0	103,360.0	35,888.9	58,727.3	80,750.0	112,347.8
Type 2 Diabetes	4,275,100	109,015.1	181,691.8	254,368.5	363,383.5	127,428.5	208,242.7	285,967.9	397,140.4
Stroke	3,473,500	31,261.5	52,102.5	72,943.5	104,205.0	32,941.5	54,557.6	75,903.7	107,427.8
Hypertension	1,342,400	32,217.6	53,696.0	75,174.4	107,392.0	37,288.9	61,018.2	83,900.0	116,730.4
**High-Fat Dairy Consumption**									
Colorectal Cancer	1,397,600	67,084.8	111,808.0	156,531.2	223,616.0	92,149.5	147,115.8	197,640.4	266,209.5
Stroke	3,473,500	46,892.3	78,153.8	109,415.3	156,307.5	50,776.7	83,811.0	116,213.8	163,672.8

*Pr* Prevalence exposure. Pr values of 15%, 25%, 35%, and 50% represent the proportion of the general population who consumes high levels of dairy

Two of the identified meta-analyses provided a statistically significant summary relative risk estimate for total milk consumption and risk of chronic disease (colorectal cancer [37] and T2D [38]); five provided significant estimates for low-fat dairy consumption (breast cancer [32], T2D [39], stroke [34], and hypertension [40]); and two provided significant estimates for high-fat dairy consumption (colorectal cancer [41] and stroke [42]) (Table 1). While a significant estimate for low-fat dairy consumption and coronary heart disease was reported in Alexander et al. [42], no estimates were available for overall CVD; thus, this estimate was not included in the analyses. The SRREs from the remaining meta-analyses were not significant; that is, their 95% confidence intervals included 1.0 and thus, they were not included in these analyses. The DALYs preventive fraction estimates based on population milk consumption of 15%, 25%, 35%, and 50% ranged from 2 2.0% for T2D to 9.5% for colorectal cancer; for population consumption of low-fat dairy products, the estimates ranged from 0.9% for stroke to 8.5% for T2D; for population consumption of high-fat dairy products, the estimates ranged from 1.4% for stroke to 16.0% for colorectal cancer. The PARP estimates for DALYs attributable to low population milk consumption ranged from 2.2% (T2D) to 10.5% (colorectal cancer); for low-fat dairy consumption, estimates ranged from 1.0% (stroke) to 9.3% (T2D); for high-fat dairy consumption, estimates ranged from 1.5% (stroke) to 19.1% (colorectal cancer) (Table 2). The number of DALYs that may be prevented by higher population consumption of milk ranged from 133,000 (colorectal cancer) to 278,000 (T2D); for low-fat dairy, the number of preventable DALYs ranged from 103,000 (breast cancer) to 363,000 (T2D); for high-fat dairy, the number of preventable DALYs ranged from 51,000 (stroke) to 92,000 (colorectal cancer) (Table 3). The number of DALYs in the US that may be attributable to lower population consumption of milk ranged from about 147,000 (colorectal cancer) to 297,000 (T2D); the number of attributable DALYs due to lower population consumption of low-fat dairy products ranged from 107,500 (stroke) to 397,000 (T2D); and for lower population consumption of high-fat dairy products, the number of attributable DALYs ranged from 164,000 (stroke) to 266,000 (colorectal cancer) (Table 3).

## Discussion

This analysis provides a novel approach for estimating the theoretical impact on the burden of disease based on dairy consumption, a modifiable dietary behavior. In such an analysis, multiple assumptions are made and must be stated clearly. First, attributable risk estimates operate under the assumption that the exposure is a cause of (or prevents) the outcome of interest. As indicated earlier, we did not perform a systematic causal assessment pertaining to dairy consumption and the selected outcomes. Rather, we assumed that increased dairy intake was associated with decreased risk of the selected outcomes based on the reported findings across the published meta-analyses which were peer reviewed publications derived from publicly funded research. Second, the meta-analyses used as the basis for this analysis quantitatively summarize data from observational studies. Thus, the prevailing methodological considerations (e.g., self-reported dietary recall, unmeasured and residual confounding, collinearity with other dietary and lifestyle factors, selection bias) when interpreting findings from nutritional epidemiology studies are relevant. A related limitation is that this analysis utilized DALYs for the US but the summary estimates from meta-analyses were based on multiple observational studies, some of which were conducted outside the US. Finally, our methodology does not take into account other risk factors for the outcomes of interest or interactions between such risk factors, and thus our results may be over-stated if the individual population attributable risks for all risk factors exceeds 100%. With these assumptions and limitations noted, our analyses were conducted to provide a methodological and analytical framework to estimate the theoretical impact of low or high dairy consumption on the burden of selected diseases in the general US population. We chose to use dairy consumption in this analysis because it is a modifiable behavior, a high proportion of the general population consumes dairy products, and there is a robust volume of published prospective cohort studies and meta-analyses of dairy consumption and chronic disease outcomes.

Our analysis was based on an exposure that has been associated with decreased risks of several chronic disease outcomes of public health interest. Furthermore, our reliance upon statistically significant findings from comprehensive meta-analyses is a conservative approach in that we only examined outcomes with consistent associations in numerous analytical epidemiologic investigations. This selection of outcomes with robust and consistent findings, despite presence of heterogeneity in some studies, adds confidence to the interpretation of patterns of results observed in our analyses. The range of prevalence exposure estimates are based on categories of dairy intake as defined by the epidemiology studies in the meta-analyses, which generally coincide with USDA dairy recommendations. Thus, the DALYs estimates based on our analyses may be considered conservative and do not represent the most extreme values of dairy intake in the population.

The concept of this analysis was based upon a publication by Doidge et al. [29], who conducted an economically-focused attributable risk analysis from an increase in consumption of dairy products. The authors applied summary relative risk estimates from recent meta-analyses of dairy consumption and various chronic health outcomes to the burden of disease and direct healthcare expenditure in Australia from 2010–2011 using the population attributable risk calculation. The health outcomes assessed were obesity, T2D, ischemic heart disease, stroke, hypertension, and osteoporosis. The authors reported that for the total of all evaluated health conditions, approximately 2 billion AUD$ could be saved in healthcare expenditures and 75,000 DALYs (estimated for the Australian population alone) could possibly be prevented by increasing dairy consumption. Though the focus of our paper was on burden of disease alone, our results for DALYS possibly prevented by increased dairy consumption are concordant with those of Doidge et al. Similar methods have been used in recent studies, including a Canadian cost-savings analysis due to increased consumption of pulses (beans, peas, and lentils) resulting in decreased complications from T2D and CVD incidence [43], an Australian study estimating the potential savings in healthcare expenditure and increased productivity due to decreased CVD and T2D prevalence with increased intake of cereal fiber [44], and a US cost-savings analysis due to increased dairy consumption resulting in decreased or increased risk of various health outcomes (T2D, stroke, hypertension, colorectal cancer, prostate cancer, Parkinson’s disease, hip fractures [45].

Though multiple meta-analyses were identified evaluating the association between dairy consumption and prostate cancer, we elected not to perform analyses for this outcome due to a lack of consistent and significant results observed in the epidemiologic literature. A meta-analysis published by Aune et al. presented an SSRE for total dairy and total prostate cancer of 1.07 (95% CI: 1.02–1.12) [46]. Huncharek et al. published a meta-analysis of dairy consumption and prostate cancer with non-significant summary results (SRRE: 1.06, 95% CI: 0.92–1.22) [47], and Gao et al. observed a marginally statistically significant summary result in their 2005 meta-analysis (SRRE: 1.11, 95% CI: 1.00–1.78) [48]. Indeed, the World Cancer Research Fund, in their 2014 evaluation of prostate cancer risk factors, considered the evidence on dairy products and prostate cancer to be “limited-suggestive” of an increase in risk [49]. Our conservative approach limited the studies included in this analysis to those with consistent evidence of a statistically significant association replicated throughout the epidemiologic literature; thus, due to the inconsistent nature of the relationship between dairy products and prostate cancer, we chose not to use the outcome of prostate cancer in our analyses.

A large volume of epidemiologic studies and reviews have been published evaluating the relationship between dairy consumption and breast cancer, colorectal cancer, CVD, and T2D. Several risk factors, protective factors and mechanisms have been postulated for the relationship between dairy consumption and the aforementioned health outcomes. Components of dairy products including calcium, vitamin D, and conjugated linoleic acid have been proposed to reduce breast cancer risk due to anti-carcinogenic properties [32, 50–53]. However, increased consumption of dairy products may represent an increase in overall saturated fat intake, which has been suggested as a possible risk factor for breast cancer [19, 20, 32, 54]. Epidemiologic evidence indicates a reduced risk of colorectal cancer with increased dairy consumption due to the bioavailability of calcium, which may reduce cell proliferation, promote cell differentiation, bind proinflammatory secondary bile acids and free ionized fatty acids, reducing their carcinogenic effects on the colorectal mucosa [9, 55–59]. However, reviews have suggested that the association may be limited to low-fat dairy products, as dietary fats have been associated with an increased risk of colorectal cancer due to increased bile acid levels in the colon [55, 59]. Additionally, dairy products increase high-density lipoprotein cholesterol which is associated with a reduced risk of CVD [60, 61]. The nutrients in dairy products such as calcium, magnesium, and milk proteins may suppress adipose tissue oxidative and inflammatory stress, thereby preventing atherosclerosis [61, 62]. Moreover, dairy products reduce the risk of hypertension, a major risk factor for CVD and stroke [5, 8, 61]. Over 100 epidemiological studies, reviews, and randomized control trials have been published describing the association between dairy intake and hypertension; the available evidence demonstrates a consistently reduced risk of hypertension due to the antihypertensive effects of calcium, potassium, lactotripeptides, and other nutrients present in dairy products [6, 13, 35, 40]. The epidemiologic evidence for T2D indicates a reduced risk associated with increased dairy consumption, based on the potential of dietary calcium, vitamin D, and magnesium to improve pancreatic β-cell function and insulin sensitivity. Another proposed mechanism for the decreased risk of T2D is the intake of dairy proteins such as whey protein, which may have an impact on overall caloric intake and glucose metabolism [8, 39, 63, 64].

## Conclusions

This analysis represents a theoretical model to estimate the impact on disease burden of a widely prevalent modifiable dietary factor. Specifically, we observed that higher dairy consumption in the US population may have a pronounced impact on reducing the number of DALYs due to breast cancer, colorectal cancer, CVD, T2D, stroke, and hypertension, and that a substantial proportion of these diseases may be attributable to low population consumption of dairy. However, these findings are based on an analytical framework of theoretical modelling and are dependent upon the reported statistical associations across published observational studies. As such, the results from our analyses are not intended to imply a causal relationship between low dairy intake and increased risk of certain chronic disease outcomes. Rather, our analyses emphasize the importance of meeting dietary guidelines as they provide a population-based extension of the findings from meta-analyses of prospective studies that indicate decreased risk of several chronic diseases of public health importance in relation to increased dairy consumption.

## Supplementary Information

**Additional file 1:** **Supplementary Table 1.** Search strings utilized to identify meta-analyses for dairy and health outcomes.

**Additional file 2:** **Supplementary Table 2.** Considered meta-analyses for the six chronic disease outcomes.

## Data Availability

All data analyzed during this study are included in this published article and its supplementary information files.

## Abbreviations

CVD: Cardiovascular disease
DALYs: Disability adjusted life years
PARPs: Population-attributable risk proportions
PF: Preventive fraction
*Pr*: Prevalence of exposure
RR: Relative risk
SRREs: Summary relative risk estimates
T2D: Type 2 diabetes
US: United States
USDA: United States Department of Agriculture
WHO: World Health Organization
YLD: Years lived with disability
YLL: Years of life lost

## References

[1] WHO: The World Health Report 2002: Reducing Risks, Promoting Healthy Life. In. Geneva: World Health Organization; 2002. 10.1080/1357628031000116808 14741909

[2] WHO: Global Health Risks: Mortality and burden of disease attributable to selected major risks. In. Geneva: World Health Organization; 2009.

[3] WHO: Global Recommendations on Physical Activity for Health. In. Geneva: World Health Organization; 2010. 26180873

[4] WHO: Global status report on noncommunicable diseases 2010. In.: World Health Organization. 2011.

[5] Soedamah-Muthu SS, Ding EL, Al-Delaimy WK, Hu FB, Engberink MF, Willett WC, Geleijnse JM (2011). Milk and dairy consumption and incidence of cardiovascular diseases and all-cause mortality: dose-response meta-analysis of prospective cohort studies. Am J Clin Nutr.

[6] Dong JY, Szeto IM, Makinen K, Gao Q, Wang J, Qin LQ, Zhao Y (2013). Effect of probiotic fermented milk on blood pressure: a meta-analysis of randomised controlled trials. Br J Nutr.

[7] Dror DK (2014). Dairy consumption and pre-school, school-age and adolescent obesity in developed countries: a systematic review and meta-analysis. Obes Rev.

[8] Elwood PC, Pickering JE, Givens DI, Gallacher JE (2010). The consumption of milk and dairy foods and the incidence of vascular disease and diabetes: an overview of the evidence. Lipids.

[9] Ralston RA, Truby H, Palermo CE, Walker KZ (2014). Colorectal cancer and nonfermented milk, solid cheese, and fermented milk consumption: a systematic review and meta-analysis of prospective studies. Crit Rev Food Sci Nutr.

[10] Godos J, Tieri M, Ghelfi F, Titta L, Marventano S, Lafranconi A, Gambera A, Alonzo E, Sciacca S, Buscemi S (2020). Dairy foods and health: an umbrella review of observational studies. Int J Food Sci Nutr.

[11] Pereira PC (2014). Milk nutritional composition and its role in human health. Nutrition.

[12] Rice BH, Quann EE, Miller GD (2013). Meeting and exceeding dairy recommendations: effects of dairy consumption on nutrient intakes and risk of chronic disease. Nutr Rev.

[13] Soedamah-Muthu SS, Verberne LD, Ding EL, Engberink MF, Geleijnse JM (2012). Dairy consumption and incidence of hypertension: a dose-response meta-analysis of prospective cohort studies. Hypertension.

[14] Kriebitzsch C, Verlinden L, Eelen G, Tan BK, Van Camp M, Bouillon R, Verstuyf A (2009). The impact of 1,25(OH)2D3 and its structural analogs on gene expression in cancer cells–a microarray approach. Anticancer Res.

[15] Huth PJ, Park KM (2012). Influence of dairy product and milk fat consumption on cardiovascular disease risk: a review of the evidence. Adv Nutr.

[16] Mensink RP, Zock PL, Kester AD, Katan MB (2003). Effects of dietary fatty acids and carbohydrates on the ratio of serum total to HDL cholesterol and on serum lipids and apolipoproteins: a meta-analysis of 60 controlled trials. Am J Clin Nutr.

[17] Siri-Tarino PW, Sun Q, Hu FB, Krauss RM (2010). Meta-analysis of prospective cohort studies evaluating the association of saturated fat with cardiovascular disease. Am J Clin Nutr.

[18] de Souza RJ, Mente A, Maroleanu A, Cozma AI, Ha V, Kishibe T, Uleryk E, Budylowski P, Schunemann H, Beyene J (2015). Intake of saturated and trans unsaturated fatty acids and risk of all cause mortality, cardiovascular disease, and type 2 diabetes: systematic review and meta-analysis of observational studies. BMJ.

[19] Boyd NF, Stone J, Vogt KN, Connelly BS, Martin LJ, Minkin S (2003). Dietary fat and breast cancer risk revisited: a meta-analysis of the published literature. Br J Cancer.

[20] Khodarahmi M, Azadbakht L (2014). The association between different kinds of fat intake and breast cancer risk in women. Int J Prev Med.

[21] USDA (2020). Dietary Guidelines for Americans 2020–2025.

[22] USDA: What We Eat in America, NHANES 2017–2018. In. Washington, D.C.: United States Department of Agriculture; 2020.

[23] USDA: Agriculture Fact Book: Profiling Food Consumption in America. In. Washington, D.C.: United States Department of Agriculture; 2003.

[24] Dietary Guidelines Advisory Committee (2020). Scientific Report of the 2020 Dietary Guidelines Advisory Committee: Advisory Report to the Secretary of Agriculture and the Secretary of Health and Human Services.

[25] WHO: Twelfth General Programme of Work: Not merely the absence of disease. In. Geneva: World Health Organization; 2014.

[26] Murray CJ, Vos T, Lozano R, Naghavi M, Flaxman AD, Michaud C, Ezzati M, Shibuya K, Salomon JA, Abdalla S (2012). Disability-adjusted life years (DALYs) for 291 diseases and injuries in 21 regions, 1990–2010: a systematic analysis for the Global Burden of Disease Study 2010. Lancet.

[27] Collaborators TUBoD (2018). The State of US Health, 1990–2016: Burden of Diseases, Injuries, and Risk Factors Among US States. JAMA.

[28] WHO (2018). WHO methods and data sources for global burden of disease estimates 2000-2016. In., vol. WHO/HIS/IER/GHE/2018.4.

[29] Doidge JC, Segal L, Gospodarevskaya E (2012). Attributable risk analysis reveals potential healthcare savings from increased consumption of dairy products. J Nutr.

[30] Greenland S, Rothman KJ, Lash TL, Rothman KJ, Greenland S, Lash TL (2008). Measures of Effect and Measures of Association. Modern Epidemiology.

[31] WHO (2018). Disease burden and mortality estimates, 2006–2016: DALYs.

[32] Dong JY, Zhang L, He K, Qin LQ (2011). Dairy consumption and risk of breast cancer: a meta-analysis of prospective cohort studies. Breast Cancer Res Treat.

[33] Schwingshackl L, Schwedhelm C, Hoffmann G, Knüppel S, Laure Preterre A, Iqbal K, Bechthold A, De Henauw S, Michels N, Devleesschauwer B (2018). Food groups and risk of colorectal cancer. Int J Cancer.

[34] Gholami F, Khoramdad M, Esmailnasab N, Moradi G, Nouri B, Safiri S, Alimohamadi Y (2017). The effect of dairy consumption on the prevention of cardiovascular diseases: a meta-analysis of prospective studies. J Cardiovasc Thorac Res.

[35] Schwingshackl L, Schwedhelm C, Hoffmann G, Knuppel S, Iqbal K, Andriolo V, Bechthold A, Schlesinger S, Boeing H (2017). Food groups and risk of hypertension: a systematic review and dose-response meta-analysis of prospective studies. Adv Nutr.

[36] Schwingshackl L, Hoffmann G, Lampousi AM, Knüppel S, Iqbal K, Schwedhelm C, Bechthold A, Schlesinger S, Boeing H (2017). Food groups and risk of type 2 diabetes mellitus: a systematic review and meta-analysis of prospective studies. Eur J Epidemiol.

[37] Jin S, Kim Y, Je Y (2020). Dairy consumption and risks of colorectal cancer incidence and mortality: a meta-analysis of prospective cohort studies. Cancer Epidemiol Biomark Prev.

[38] Tian S, Xu Q, Jiang R, Han T, Sun C, Na L (2017). Dietary protein consumption and the risk of type 2 diabetes: a systematic review and meta-analysis of cohort studies. Nutrients.

[39] Aune D, Norat T, Romundstad P, Vatten LJ (2013). Dairy products and the risk of type 2 diabetes: a systematic review and dose-response meta-analysis of cohort studies. Am J Clin Nutr.

[40] Ralston RA, Lee JH, Truby H, Palermo CE, Walker KZ (2012). A systematic review and meta-analysis of elevated blood pressure and consumption of dairy foods. J Hum Hypertens.

[41] Barrubés L, Babio N, Becerra-Tomás N, Rosique-Esteban N, Salas-Salvadó J (2019). Association between dairy product consumption and colorectal cancer risk in adults: a systematic review and meta-analysis of epidemiologic studies. Adv Nutr.

[42] Alexander DD, Bylsma LC, Vargas AJ, Cohen SS, Doucette A, Mohamed M, Irvin SR, Miller PE, Watson H, Fryzek JP (2016). Dairy consumption and CVD: a systematic review and meta-analysis. Br J Nutr.

[43] Abdullah MMH, Marinangeli CPF, Jones PJH, Carlberg JG (2017). Canadian Potential Healthcare and Societal Cost Savings from Consumption of Pulses: A Cost-Of-Illness Analysis. Nutrients.

[44] Fayet-Moore F, George A, Cassettari T, Yulin L, Tuck K, Pezzullo L (2018). Healthcare Expenditure and Productivity Cost Savings from Reductions in Cardiovascular Disease and Type 2 Diabetes Associated with Increased Intake of Cereal Fibre among Australian Adults: A Cost of Illness Analysis. Nutrients.

[45] Scrafford CG, Bi X, Multani JK, Murphy MM, Schmier JK, Barraj LM (2020). Health Care Costs and Savings Associated with Increased Dairy Consumption among Adults in the United States. Nutrients.

[46] Aune D, Navarro Rosenblatt DA, Chan DS, Vieira AR, Vieira R, Greenwood DC, Vatten LJ, Norat T (2015). Dairy products, calcium, and prostate cancer risk: a systematic review and meta-analysis of cohort studies. Am J Clin Nutr.

[47] Huncharek M, Muscat J, Kupelnick B (2008). Dairy products, dietary calcium and vitamin D intake as risk factors for prostate cancer: a meta-analysis of 26,769 cases from 45 observational studies. Nutr Cancer.

[48] Gao X, LaValley MP, Tucker KL (2005). Prospective studies of dairy product and calcium intakes and prostate cancer risk: a meta-analysis. J Natl Cancer Inst.

[49] World Cancer Research Fund/American Institute for Cancer Research. Continuous Update Project Report 2018. Diet, nutrition, physical activity, and prostate cancer. Available at https://dietandcancerreport.org.

[50] Chen P, Hu P, Xie D, Qin Y, Wang F, Wang H (2010). Meta-analysis of vitamin D, calcium and the prevention of breast cancer. Breast Cancer Res Treat.

[51] Kelley NS, Hubbard NE, Erickson KL (2007). Conjugated linoleic acid isomers and cancer. J Nutr.

[52] Michels KB, Mohllajee AP, Roset-Bahmanyar E, Beehler GP, Moysich KB (2007). Diet and breast cancer: a review of the prospective observational studies. Cancer.

[53] Moorman PG, Terry PD (2004). Consumption of dairy products and the risk of breast cancer: a review of the literature. Am J Clin Nutr.

[54] Thiebaut AC, Kipnis V, Chang SC, Subar AF, Thompson FE, Rosenberg PS, Hollenbeck AR, Leitzmann M, Schatzkin A (2007). Dietary fat and postmenopausal invasive breast cancer in the National Institutes of Health-AARP Diet and Health Study cohort. J Natl Cancer Inst.

[55] Aune D, Lau R, Chan DS, Vieira R, Greenwood DC, Kampman E, Norat T (2012). Dairy products and colorectal cancer risk: a systematic review and meta-analysis of cohort studies. Ann Oncol.

[56] Green CJ, de Dauwe P, Boyle T, Tabatabaei SM, Fritschi L, Heyworth JS (2014). Tea, coffee, and milk consumption and colorectal cancer risk. J Epidemiol.

[57] Keum N, Aune D, Greenwood DC, Ju W, Giovannucci EL (2014). Calcium intake and colorectal cancer risk: dose-response meta-analysis of prospective observational studies. Int J Cancer.

[58] Murphy N, Norat T, Ferrari P, Jenab M, Bueno-de-Mesquita B, Skeie G, Olsen A, Tjonneland A, Dahm CC, Overvad K (2013). Consumption of dairy products and colorectal cancer in the European Prospective Investigation into Cancer and Nutrition (EPIC). PLoS One.

[59] Norat T, Riboli E (2003). Dairy products and colorectal cancer. A review of possible mechanisms and epidemiological evidence. Eur J Clin Nutr.

[60] Astrup A (2014). Yogurt and dairy product consumption to prevent cardiometabolic diseases: epidemiologic and experimental studies. Am J Clin Nutr.

[61] German JB, Gibson RA, Krauss RM, Nestel P, Lamarche B, van Staveren WA, Steijns JM, de Groot LC, Lock AL, Destaillats F (2009). A reappraisal of the impact of dairy foods and milk fat on cardiovascular disease risk. Eur J Nutr.

[62] Lin PH, Yeh WT, Svetkey LP, Chuang SY, Chang YC, Wang C, Pan WH (2013). Dietary intakes consistent with the DASH dietary pattern reduce blood pressure increase with age and risk for stroke in a Chinese population. Asia Pac J Clin Nutr.

[63] Gao D, Ning N, Wang C, Wang Y, Li Q, Meng Z, Liu Y, Li Q (2013). Dairy products consumption and risk of type 2 diabetes: systematic review and dose-response meta-analysis. PLoS One.

[64] Hirahatake KM, Slavin JL, Maki KC, Adams SH (2014). Associations between dairy foods, diabetes, and metabolic health: potential mechanisms and future directions. Metabolism.

