# Supplementary material for: Theoretical attributable risk analysis and Disability Adjusted Life Years (DALYs) based on increased dairy consumption
Source: BMC Public Health. 2022 Aug 27;22:1625. doi: 10.1186/s12889-022-14042-7 (PMC9420283; doi:10.1186/s12889-022-14042-7)
Supplement: Supplementary file 1 — Additional file 1: Supplementary Table 1. Search strings utilized to identify meta-analyses for dairy and health outcomes. [file 12889_2022_14042_MOESM1_ESM.docx]

**Supplementary Table 1.** Search strings utilized to identify meta-analyses for dairy and health outcomes

| **Outcome** | **Database** | **Search String** |
| --- | --- | --- |
| Breast Cancer | PubMed | (((breast cancer[MeSH Terms]) AND ((dairy products[MeSH Terms]) OR (dairy product[MeSH Terms]) OR (products, dairy[MeSH Terms])))) AND Review[Filter] |
|  | PubMed | (((breast cancer[MeSH Terms]) AND ((dairy products[MeSH Terms]) OR (dairy product[MeSH Terms]) OR (products, dairy[MeSH Terms])))) AND meta-analysis |
|  | Web of Science | Searched for studies citing Dong (2011)[1] |
| Colorectal Cancer | PubMed | (((colorectal cancer[MeSH Terms]) AND ((dairy products[MeSH Terms]) OR (dairy product[MeSH Terms]) OR (products, dairy[MeSH Terms])))) AND Review[Filter] |
|  | PubMed | ((((colorectal cancer[MeSH Terms]) AND ((dairy products[MeSH Terms]) OR (dairy product[MeSH Terms]) OR (products, dairy[MeSH Terms])))) AND meta-analysis |
|  | Web of Science | Searched for studies citing Aune (2012)[2] |
| Hypertension | PubMed | ((((dairy products[MeSH Terms]) OR (products, dairy[MeSH Terms]) OR (dairy product[MeSH Terms])) AND ((hypertension[MeSH Terms]) OR (blood pressure[MeSH Terms])))) AND Review[Filter] |
|  | PubMed | (((dairy products[MeSH Terms]) OR (products, dairy[MeSH Terms]) OR (dairy product[MeSH Terms])) AND ((hypertension[MeSH Terms]) OR (blood pressure[MeSH Terms])) AND meta-analysis) |
|  | Web of Science | Searched for studies citing Schwingshackl (2017)[3] |
| CVD | PubMed | ((((cardiovascular disease[MeSH Terms]) OR (artery disease, coronary[MeSH Terms]) OR (acute coronary syndrome[MeSH Terms]) OR (arterioscleroses, coronary[MeSH Terms]) OR (heart[MeSH Terms]) OR ("stroke"[MeSH Terms]) OR ("cerebrovascular disorders"[MeSH Terms]) OR (disease, peripheral arterial[MeSH Terms]) OR (cerebrovascular accident[MeSH Terms])) AND ((dairy products[MeSH Terms]) OR (products, dairy[MeSH Terms]) OR (dairy product[MeSH Terms])))) AND Review[Filter] |
|  | PubMed | ((cardiovascular disease[MeSH Terms]) OR (artery disease, coronary[MeSH Terms]) OR (acute coronary syndrome[MeSH Terms]) OR (arterioscleroses, coronary[MeSH Terms]) OR (heart[MeSH Terms]) OR ("stroke"[MeSH Terms]) OR ("cerebrovascular disorders"[MeSH Terms]) OR (disease, peripheral arterial[MeSH Terms]) OR (cerebrovascular accident[MeSH Terms])) AND ((dairy products[MeSH Terms]) OR (products, dairy[MeSH Terms]) OR (dairy product[MeSH Terms])) AND meta-analysis |
|  | Web of Science | Searched for studies citing Gholami (2017)[4] |
| Type 2 Diabetes | PubMed | (((((dairy products[MeSH Terms]) OR products, dairy[MeSH Terms]) AND type 2 diabetes mellitus[MeSH Terms])) AND Review[Filter] |
|  | PubMed | ((dairy products[MeSH Terms]) OR products, dairy[MeSH Terms]) AND type 2 diabetes mellitus[MeSH Terms] AND meta-analysis |
|  | Web of Science | Searched for studies citing Aune (2013)[5] |
| Prostate cancer | PubMed | ((((((prostate cancer[MeSH Terms]) AND ((dairy products[MeSH Terms]) OR (dairy product[MeSH Terms]) OR (products, dairy[MeSH Terms])))))) AND Review[Filter] |
|  | PubMed | ((((prostate cancer[MeSH Terms]) AND ((dairy products[MeSH Terms]) OR (dairy product[MeSH Terms]) OR (products, dairy[MeSH Terms])))) AND meta-analysis |
|  | Web of Science | Searched for studies citing Aune (2015)[6] |
| Bone fractures | PubMed | ((((bone fractures[MeSH Terms]) AND ((dairy products[MeSH Terms]) OR (dairy product[MeSH Terms]) OR (products, dairy[MeSH Terms])))) AND Review[Filter] |
|  | PubMed | ((bone fractures[MeSH Terms]) AND ((dairy products[MeSH Terms]) OR (dairy product[MeSH Terms]) OR (products, dairy[MeSH Terms])) AND meta analysis) |
| Obesity | PubMed | obesity AND (dairy consumption) AND meta-analysis |
|  | PubMed | obesity[MeSH Terms]) AND ((dairy products[MeSH Terms]) OR (dairy product[MeSH Terms]) OR (products, dairy[MeSH Terms])) AND meta-analysis |

References

1. Dong JY, Zhang L, He K, Qin LQ: **Dairy consumption and risk of breast cancer: a meta-analysis of prospective cohort studies**. *Breast Cancer Res Treat* 2011, **127**(1):23-31.

2. Aune D, Lau R, Chan DS, Vieira R, Greenwood DC, Kampman E, Norat T: **Dairy products and colorectal cancer risk: a systematic review and meta-analysis of cohort studies**. *Ann Oncol* 2012, **23**(1):37-45.

3. Schwingshackl L, Schwedhelm C, Hoffmann G, Knuppel S, Iqbal K, Andriolo V, Bechthold A, Schlesinger S, Boeing H: **Food Groups and Risk of Hypertension: A Systematic Review and Dose-Response Meta-Analysis of Prospective Studies**. *Adv Nutr* 2017, **8**(6):793-803.

4. Gholami F, Khoramdad M, Esmailnasab N, Moradi G, Nouri B, Safiri S, Alimohamadi Y: **The effect of dairy consumption on the prevention of cardiovascular diseases: A meta-analysis of prospective studies**. *J Cardiovasc Thorac Res* 2017, **9**(1):1-11.

5. Aune D, Norat T, Romundstad P, Vatten LJ: **Dairy products and the risk of type 2 diabetes: a systematic review and dose-response meta-analysis of cohort studies**. *Am J Clin Nutr* 2013, **98**(4):1066-1083.

6. Aune D, Navarro Rosenblatt DA, Chan DS, Vieira AR, Vieira R, Greenwood DC, Vatten LJ, Norat T: **Dairy products, calcium, and prostate cancer risk: a systematic review and meta-analysis of cohort studies**. *Am J Clin Nutr* 2015, **101**(1):87-117.
