# Supplementary material for: Theoretical attributable risk analysis and Disability Adjusted Life Years (DALYs) based on increased dairy consumption
Source: BMC Public Health. 2022 Aug 27;22:1625. doi: 10.1186/s12889-022-14042-7 (PMC9420283; doi:10.1186/s12889-022-14042-7)
Supplement: Supplementary file 2 — Additional file 2: Supplementary Table 2. Considered meta-analyses for the six chronic disease outcomes. [file 12889_2022_14042_MOESM2_ESM.docx]

**Supplementary Table 2.** Considered meta-analyses for the six chronic disease outcomes

| **Source** | **Study Designs Included in Analysis** | **Studies Included in Analysis (n)** | **Analysis Type** | **RR** | **95% CI** | **P-value for Heterogeneity; I^2^ value** |
| --- | --- | --- | --- | --- | --- | --- |
| ***Breast Cancer*** | | | | | | |
| **Total Dairy** | | | | | | |
| **Dong (2011)[1]** | **Prospective Cohort** | **10** | **High vs. Low** | **0.85** | **0.76-0.95** | **0.012; 54.5%** |
| **Total Milk** | | | | | | |
| Dong (2011)[1] | Prospective Cohort | 12 | High vs. Low | 0.90 | 0.80-1.02 | 0.003; 59.7% |
| Chen (2019)[2] | Case-control | 8 | High vs. Low | 0.95 | 0.80-1.13 | 0.57, NR |
| **Low-Fat Dairy** | | | | | | |
| **Dong (2011)[1]** | **Prospective Cohort** | **4** | **High vs. Low** | **0.84** | **0.73-0.96** | **0.07; 53.7%** |
| **High-Fat Dairy** | | | | | | |
| Dong (2011)[1] | Prospective Cohort | 4 | High vs. Low | 0.99 | 0.85-1.15 | 0.04; 61.3% |
| ***Colorectal Cancer*** | | | | | | |
| **Total Dairy** | | | | | | |
| Aune (2012)[3] | Prospective Cohort | 12 | High vs. Low | 0.81 | 0.74-0.90 | 0.06; 42% |
| Barrubes (2019)[4] | Prospective Cohort | 8 | High vs. Low | 0.80 | 0.70-0.91 | 0.08; 45% |
| Jin (2020)[5] | Prospective Cohort | 14 | High vs. Low | 0.79 | 0.74-0.85 | 0.07; 36.8% |
| **Schwingshakl (2018)[6]** | **Cohort, case-cohort, nested case-control, follow-ups of RCTs (all prospective)** | **18** | **High vs. Low** | **0.83** | **0.76-0.89** | **<0.001; 61%** |
| **Total Milk** | | | | | | |
| Aune (2012)[3] | Prospective Cohort | 10 | High vs. Low | 0.83 | 0.74-0.93 | 0.31; 14% |
| Barrubes (2019)[4] | Prospective Cohort | 9 | High vs. Low | 0.82 | 0.76-0.88 | 0.42; 2% |
| **Jin (2020)[5]** | **Prospective Cohort** | **20** | **High vs Low** | **0.81** | **0.76-0.86** | **0.138; 23.6%** |
| **Low-Fat Dairy** | | | | | | |
| Aune (2012)[3] | Prospective Cohort | 2 | High vs. Low | 0.97 | 0.74-1.28 | 0.78; 0.0% |
| Barrubes (2019)[4] | Prospective Cohort | 2 | High vs. Low | 0.91 | 0.79-1.06 | 0.15; 52% |
| **High-Fat Dairy** | | | | | | |
| Aune (2012)[3] | Prospective Cohort | 3 | High vs. Low | 0.74 | 0.53-1.02 | 0.16; 45.0% |
| **Barrubes (2019)[4]** | **Prospective Cohort** | **2** | **High vs. Low** | **0.68** | **0.53-0.87** | **0.06; 71%** |
| ***Type 2 Diabetes*** | | | | | | |
| **Total Dairy** | | | | | | |
| Aune (2013)[7] | Cohort | 14 | High vs. Low | 0.89 | 0.82-0.96 | 0.05; 42.1% |
| Mishali (2019)[8] | Population-based cohort | 16 | High vs. Low | 0.90 | 0.83-0.96 | 0.01; 51% |
| Tian (2017)[9] | Cohort | 11 | High vs. Low | 0.89 | 0.84-0.94 | 0.03; 48.8% |
| Khoramdad (2017)[10] | Prospective cohort, case cohort | 13 | High vs. Low | 0.88 | 0.80-0.96 | NR; 94.9% |
| **Schwingshackl (2017)[11]** | **Cohort, case-control, nested case-control, follow-up of RCTs** | **21** | **High vs. Low** | **0.91** | **0.85-0.97** | **<0.0001; 63%** |
| **Total Milk** | | | | | | |
| Aune (2013)[7] | Cohort | 7 | High vs. Low | 0.87 | 0.70-1.07 | 0.002; 70.5% |
| **Tian (2017)[9]** | **Cohort** | **7** | **High vs. Low** | **0.87** | **0.78-0.96** | **0.01; 52.2%** |
| **Low-Fat Dairy** | | | | | | |
| **Aune (2013)[7]** | **Cohort** | **9** | **High vs. Low** | **0.83** | **0.76-0.90** | **0.67; 0%** |
| Khoramdad (2017)[10] | Prospective cohort, case cohort | 4 | High vs. Low | 0.81 | 0.68-0.96 | 0.033; 65.6% |
| **High-Fat Dairy** | | | | | | |
| Aune (2013)[7] | Cohort | 8 | High vs. Low | 0.96 | 0.87-1.06 | 0.31; 15.8% |
| Khoramdad (2017)[10] | Prospective cohort, case cohort | **3** | High vs. Low | 0.98 | 0.78-1.24 | 0.116; 53.6% |
| ***Cardiovascular Disease*** | | | | | | |
| **Total Dairy** | | | | | | |
| Alexander (2016)[12] | Prospective cohort | 4 | High vs. Low | 0.88 | 0.75-1.04 | 0.076; 52.7% |
| Mishali (2019)[8] | Population-based cohort | 13 | High vs. Low | 0.94 | 0.89-0.99 | 0.024; 48.8% |
| **Gholami (2017)[13]** | **Prospective cohort** | **10** | **High vs. Low** | **0.90** | **0.81-0.99** | **0.009; 55.8%** |
| **Total Milk** | | | | | | |
| Alexander (2016)[12] | Prospective cohort | 4 | High vs. Low | 0.94 | 0.86-1.03 | 0.167; 38.1% |
| **Low-Fat Dairy** | | | | | | |
| Alexander (2016)[12] | Prospective cohort | 4 | High vs. Low  (CHD*) | 0.90 | 0.82-0.98 | 0.991; 0.0% |
| Gholami (2017)[13] | Prospective cohort | 11 | High vs. Low (CHD*) | 1.01 | 0.94-1.09 | 0.00; 62.6% |
| **High-Fat Dairy** | | | | | | |
| Alexander (2016)[12] | Prospective cohort | 4 | High vs. Low  (CHD*) | 1.05 | 0.93-1.19 | 0.237; 29.3% |
| Gholami (2017)[13] | Prospective cohort | 11 | High vs. Low (CHD*) | 0.98 | 0.94-1.01 | 0.41; 2.4% |
| ***Stroke*** | | | | | | |
| **Total Dairy** | | | | | | |
| Alexander (2016)[12] | Prospective cohort | 7 | High vs. Low | 0.91 | 0.83-0.99 | 0.072; 44.5% |
| Bechthold (2019)[14] | Prospective design (cohort, case-control, nested case-control, follow-up of RCTs) | 12 | High vs. Low | 0.96 | 0.90-1.01 | 0.05; 43% |
| **Gholami (2017)[13]** | **Prospective cohort** | **16** | **High vs. Low** | **0.88** | **0.82-0.95** | **0.000; 63.1%** |
| **Total Milk** | | | | | | |
| Alexander (2016)[12] | Prospective cohort | 7 | High vs. Low | 0.90 | 0.79-1.02 | <0.001; 79.6% |
| **Low-Fat Dairy** | | | | | | |
| Alexander (2016)[12] | Prospective cohort | 3 | High vs. Low | 0.90 | 0.83-0.96 | 0.914; 0.0% |
| Bechthold (2019)[14] | Prospective design (cohort, case-control, nested case-control, follow-up of RCTs) | 7 | High vs. Low | 0.96 | 0.90-1.03 | NR; 42% |
| **Gholami (2017)[13]** | **Prospective cohort** | **9** | **High vs. Low** | **0.94** | **0.90-0.98** | **0.61; 0.0%** |
| **High-Fat Dairy** | | | | | | |
| **Alexander (2016)[12]** | **Prospective cohort** | **3** | **High vs. Low** | **0.91** | **0.84-0.99** | **0.882; 0.0%** |
| Bechthold (2019)[14] | Prospective design (cohort, case-control, nested case-control, follow-up of RCTs) | 7 | High vs. Low | 1.01 | 0.96-1.06 | NR; 9% |
| Gholami (2017)[13] | Prospective cohort | 9 | High vs. Low | 0.95 | 0.91-1.00 | 0.61; 0.0% |
| ***Hypertension*** | | | | | | |
| **Total Dairy** | | | | | | |
| Ralston (2012)[15] | Prospective cohort | 5 | High vs. Low | 0.87 | 0.81-0.94 | NR; 0% |
| **Schwingshackl (2017)[16]** | **Cohort, case-control, nested case-control, follow-up of RCTs** | **9** | **High vs. Low** | **0.89** | **0.86-0.93** | **0.65; 0%** |
| **Low-Fat Dairy** | | | | | | |
| **Ralston (2012)[15]** | **Prospective cohort** | **4** | **High vs. Low** | **0.84** | **0.74-0.95** | **NR; 38%** |
| **High-Fat Dairy** | | | | | | |
| Ralston (2012)[15] | Prospective cohort | 4 | High vs. Low | 1.00 | 0.89-1.11 | NR; 27% |

CHD, coronary heart disease; NR, not reported.

*No meta-analytic estimates reported low-fat or high-fat dairy for overall cardiovascular disease, only for the subgroup of CHD.

Bolded studies were selected for the computations performed in this work.

References

1. Dong JY, Zhang L, He K, Qin LQ: **Dairy consumption and risk of breast cancer: a meta-analysis of prospective cohort studies**. *Breast Cancer Res Treat* 2011, **127**(1):23-31.

2. Chen L, Li M, Li H: **Milk and yogurt intake and breast cancer risk: A meta-analysis**. *Medicine (Baltimore)* 2019, **98**(12):e14900.

3. Aune D, Lau R, Chan DS, Vieira R, Greenwood DC, Kampman E, Norat T: **Dairy products and colorectal cancer risk: a systematic review and meta-analysis of cohort studies**. *Ann Oncol* 2012, **23**(1):37-45.

4. Barrubés L, Babio N, Becerra-Tomás N, Rosique-Esteban N, Salas-Salvadó J: **Association Between Dairy Product Consumption and Colorectal Cancer Risk in Adults: A Systematic Review and Meta-Analysis of Epidemiologic Studies**. *Adv Nutr* 2019, **10**(suppl_2):S190-s211.

5. Jin S, Kim Y, Je Y: **Dairy Consumption and Risks of Colorectal Cancer Incidence and Mortality: A Meta-analysis of Prospective Cohort Studies**. *Cancer Epidemiology Biomarkers &amp; Prevention* 2020, **29**(11):2309-2322.

6. Schwingshackl L, Schwedhelm C, Hoffmann G, Knüppel S, Laure Preterre A, Iqbal K, Bechthold A, De Henauw S, Michels N, Devleesschauwer B *et al*: **Food groups and risk of colorectal cancer**. *Int J Cancer* 2018, **142**(9):1748-1758.

7. Aune D, Norat T, Romundstad P, Vatten LJ: **Dairy products and the risk of type 2 diabetes: a systematic review and dose-response meta-analysis of cohort studies**. *Am J Clin Nutr* 2013, **98**(4):1066-1083.

8. Mishali M, Prizant-Passal S, Avrech T, Shoenfeld Y: **Association between dairy intake and the risk of contracting type 2 diabetes and cardiovascular diseases: a systematic review and meta-analysis with subgroup analysis of men versus women**. *Nutr Rev* 2019, **77**(6):417-429.

9. Tian S, Xu Q, Jiang R, Han T, Sun C, Na L: **Dietary Protein Consumption and the Risk of Type 2 Diabetes: A Systematic Review and Meta-Analysis of Cohort Studies**. *Nutrients* 2017, **9**(9).

10. Khoramdad MA, Yousef; Safiri, Saeed; Pakzad, Reza; Shakiba, Ebrahim; Shafiei, Jabbar; Firouzi, Alireza: **Dairy products consumption and risk of type 2 diabetes: a systematic review and meta-analysis of prospective cohort studies**. *Iranian Red Crescent Medical Journal (IRCMJ)* 2017, **19**(7):-.

11. Schwingshackl L, Hoffmann G, Lampousi AM, Knüppel S, Iqbal K, Schwedhelm C, Bechthold A, Schlesinger S, Boeing H: **Food groups and risk of type 2 diabetes mellitus: a systematic review and meta-analysis of prospective studies**. *Eur J Epidemiol* 2017, **32**(5):363-375.

12. Alexander DD, Bylsma LC, Vargas AJ, Cohen SS, Doucette A, Mohamed M, Irvin SR, Miller PE, Watson H, Fryzek JP: **Dairy consumption and CVD: a systematic review and meta-analysis**. *Br J Nutr* 2016, **115**(4):737-750.

13. Gholami F, Khoramdad M, Esmailnasab N, Moradi G, Nouri B, Safiri S, Alimohamadi Y: **The effect of dairy consumption on the prevention of cardiovascular diseases: A meta-analysis of prospective studies**. *J Cardiovasc Thorac Res* 2017, **9**(1):1-11.

14. Bechthold A, Boeing H, Schwedhelm C, Hoffmann G, Knüppel S, Iqbal K, De Henauw S, Michels N, Devleesschauwer B, Schlesinger S *et al*: **Food groups and risk of coronary heart disease, stroke and heart failure: A systematic review and dose-response meta-analysis of prospective studies**. *Crit Rev Food Sci Nutr* 2019, **59**(7):1071-1090.

15. Ralston RA, Lee JH, Truby H, Palermo CE, Walker KZ: **A systematic review and meta-analysis of elevated blood pressure and consumption of dairy foods**. *J Hum Hypertens* 2012, **26**(1):3-13.

16. Schwingshackl L, Schwedhelm C, Hoffmann G, Knuppel S, Iqbal K, Andriolo V, Bechthold A, Schlesinger S, Boeing H: **Food Groups and Risk of Hypertension: A Systematic Review and Dose-Response Meta-Analysis of Prospective Studies**. *Adv Nutr* 2017, **8**(6):793-803.
